# Lipidomic Approaches towards Deciphering Glycolipids from Microalgae as a Reservoir of Bioactive Lipids

**DOI:** 10.3390/md14050101

**Published:** 2016-05-19

**Authors:** Elisabete da Costa, Joana Silva, Sofia Hoffman Mendonça, Maria Helena Abreu, Maria Rosário Domingues

**Affiliations:** 1 Centro de Espectrometria de Massa, Departamento de Química & QOPNA, Universidade de Aveiro, Campus Universitário de Santiago, 3810-193 Aveiro, Portugal; elisabetecosta@ua.pt; 2 Allmicroalgae—Natural Products S.A., Avenida das Forças Armadas, 125, 7º piso, 1600-079 Lisboa, Portugal; jglaranjeira2@gmail.com (J.S.); sofia.mendonca@allmicroalgae.com (S.H.M.); 3 ALGAplus-Produção e Comercialização de Algas e Derivados, Lda., 3830-196 Ílhavo, Portugal; htabreu@algaplus.pt

**Keywords:** lipidomic, glycolipids, microalgae, fatty acids, mass spectrometry, bioprospection

## Abstract

In recent years, noteworthy research has been performed around lipids from microalgae. Among lipids, glycolipids (GLs) are quite abundant in microalgae and are considered an important source of fatty acids (FAs). GLs are rich in 16- and 18-carbon saturated and unsaturated fatty acids and often contain polyunsaturated fatty acids (PUFAs) like *n*-3 α-linolenic (ALA 18:3), eicosapentaenoic (EPA, 20:5) and docosahexaenoic (DHA, 22:6). GLs comprise three major classes: monogalactosyldiacyl glycerolipids (MGDGs), digalactosyl diacylglycerolipids (DGDGs) and sulfoquinovosyl diacylglycerolipids (SQDGs), whose composition in FA directly depends on the growth conditions. Some of these lipids are high value-added compounds with antitumoral, antimicrobial and anti-inflammatory activities and also with important nutritional significance. To fully explore GLs’ bioactive properties it is necessary to fully characterize their structure and to understand the relation between the structure and their biological properties, which can be addressed using modern mass spectrometry (MS)-based lipidomic approaches. This review will focus on the up-to-date FA composition of GLs identified by MS-based lipidomics and their potential as phytochemicals.

## 1. Introduction

Microalgae are a group of unicellular photosynthetic microscopic organisms classified as prokaryotic (bacteria, Kingdom Monera) and eukaryotic (Kingdom Protista) further distributed into several divisions, depending on their pigmentation and physiology [1,2]. Among the most common divisions there are the prokaryotic Cyanophyceae known as cyanobacteria, the eukaryotic Chlorophyceae (green algae) and the Bacillariophyceae (Heterokont) [1,2,3]. Microalgae are widely found in aquatic and terrestrial habitats mostly due to their particular versatility in adjusting to distinct growth conditions [4]. Due to this capacity, microalgae are currently cultivated on a large scale, both in artificial or natural ponds and in photoreactors (PBR), representing an important group of organisms for biotechnological exploitation with an important impact in the food and pharmaceutical industries and public health [5,6,7]. The microalgae-based product market has reached a size of about 8000 ton/year of dry matter, generating an annual turnover of U.S. $ 4 × 10^8^/year [5]. The biomass produced by several species like *Dunaliella*, *Arthrospira* (*Spirulina*) and *Chlorella* is already being marketed in various forms designed for human nutrition, either incorporated into foods and beverages or as a healthy nutritional supplement, rich in carbohydrates, proteins, vitamins, minerals, pigments and lipids [6,7,8].

Nowadays, due to the high content of lipids, there has been increasing attention in the use of microalgae as a source of lipids with various commercial applications, for example, as supplementary food or in the chemical, pharmaceutical and cosmetic industries [8,9]. Lipids of some microalgae species are enriched in valuable polyunsaturated fatty acids (PUFAs) that are mainly esterified to other lipids. Such lipids can be neutral/non-polar lipids like mono, di- and tri-acylglycerides, or polar lipids including phospholipids and glycolipids [10,11,12]. Glycolipids (GLs) represent a less studied class of lipids that captured the growing interest of researchers. They are located in the membrane of chloroplasts and thylakoids, and are important signal and regulatory molecules [11,12,13]. The most abundant glycolipids found in microalgae are monogalactosyl diacylglycerols (MGDGs), digalactosyl diacylglycerols (DGDGs) and sulfoquinovosyl diacylglycerols (SQDGs), which are rich in PUFAs, namely linoleic (LA, 18:2*n*-6), arachidonic (ARA, 20:4*n*-6), *α*-linolenic (ALA, 18:3*n*-3), eicosapentaenoic (EPA, 20:5*n*-3) and docosahexaenoic (DHA, 22:6*n*-3) fatty acids [11,14,15]. GLs from microalgae are considered an important nutritional source of *n*-3 fatty acids with beneficial health effects to humans, contributing to the balance between *n*-3 and *n*-6 FAs (fatty acids). In addition, some GLs extracted from microalgae and macroalgae possess biological activities such as antifungal, antiviral and antitumoral properties [16,17,18,19,20,21]. They also present health benefits as anti-inflammatory and with antimicrobial properties [17]. The growing resistance of pathogenic bacteria against commercially available antimicrobial drugs turns the search for new microbial substances into one of increasing importance. The biological activity of GLs is dependent on the sugar moiety and acyl chains, however the specific relation of structure–activity remains unknown [22].

The detailed characterization of GLs structures and profile from microalgae is not an easy task due to the high number of different GLs molecular species present in the full lipidome. Additionally, the changes in the content and composition of GLs, namely the composition in fatty acids that may occur within the same microalgae species, depends on the growth and environmental conditions such as light, salinity, temperature, contaminants parameters and nutrient availability, increasing the large chemical diversity and complexity of the structures [10,15,23].

Until now, few studies focusing on the glycolipidome of microalgae have been performed due to the complexity of the full lipidome [14,15,24,25,26,27,28]. Considering the recognized biological role of some glycolipids, more efforts are needed to identify and evaluate the structural features of GLs and the correspondent signature of each microalgae. To overcome this challenge, MS-based approaches represent a new emerging technology that will provide new insights which are capable of affording a better understanding of the mechanisms involved in GLs activities. This work presents a succinct review of the studies, including MS-based approaches, towards deciphering the structure of glycolipids isolated from microalgae and their potential bioactive properties.

## 2. The Structure of Glycolipids

**Figure 1:**
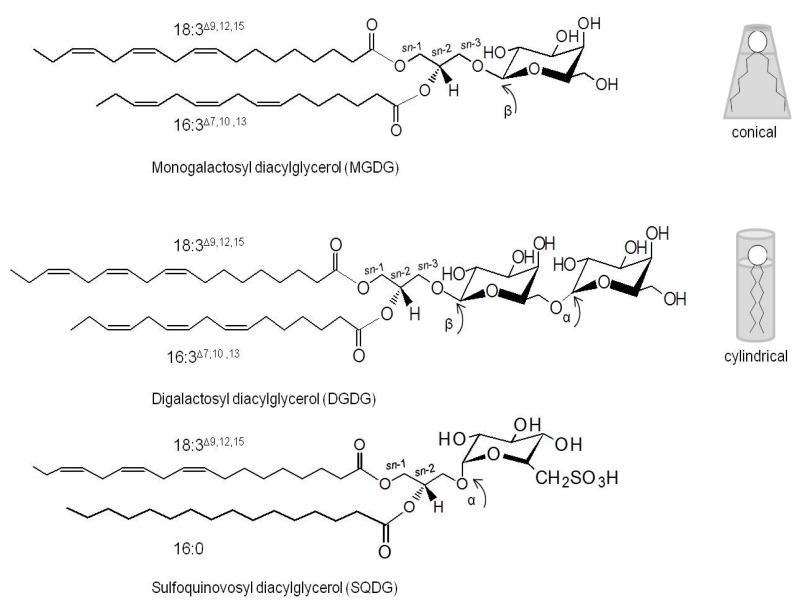
Main glycerolipid classes conserved in photosynthetic membranes of algae: MGDG, monogalactosyl diacylglycerol; DGDG, diagalactosyl diacylglycerol; SQDG, sulfoquinovosyl diacylglycerol; R_1_ and R_2_ represent fatty acyl chains. In the membrane structure of chloroplasts, MDGD tends to adapt a conical shape; DGDG and SQDG tend to adapt cylindrical shapes.

Glycolipids in microalgae are glyceroglycolipids that have a glycerol backbone which anchors one or two acyl chains esterified (R_1_ and R_2_) at positions *sn*-1 and *sn*-2, and a sugar moiety linked at position *sn*-3 in a β-anomeric linkage (Figure 1) [29]. Depending on the composition of the glycosidic head group, the GLs can be classified as monogalactosyl diacylglycerol (MGDG), digalactosyl diacylglycerol (DGDG) and sulfoquinovosyl diacylglycerol (SQDG) [10,13]. Each GLs class can accommodate several molecular species depending on the fatty acids (FA) that are linked to *sn*-1 and *sn*-2 positions of the glycerol backbone. FAs with different chain lengths and degrees of unsaturation can be distributed in *sn*-1 and *sn*-2 positions of glycerol [29,30]. The properties of GLs directly depend on the sugar moiety and on the length, degree of unsaturation and regiospecificity of the two acyl chains in the *sn*-1 and *sn*-2 positions of glycerol [22].

The predominant GLs in microalgae are the neutral and uncharged galactosylglycerides, MGDGs and DGDGs. MGDG contains one galactose β-anomeric linked to the *sn*-3 position of glycerol backbone corresponding to 1,2-diacyl-3-*O*-(β-d-galactopyranosyl)-*sn*-glycerol. DGDG is characterized by a terminal α-galactose moiety (1→6) linked to the inner β-galactose residue, also named 1,2-diacyl-3-*O*-(α-galactosyl-(1,6)-*O*-β-d-galactopyranosyl-*sn*-glycerol. MGDG and DGDG molecular species are characterized by their high content of PUFA, mostly 16- and 18-carbon *n*-3 fatty acids, and often contain very long chain polyunsaturated acids, with more than 20 carbon atoms and more than three double bonds such as eicosapentaenoic acid (EPA) [29,30]. MGDG tends to adapt a conical shape if the fatty acids are highly unsaturated (non-bilayer-forming lipid). Meanwhile, MGDG containing saturated fatty acids preferably adapt to a cylindrical form, while DGDG has a more cylindrical shape and forms lipid bilayers (Figure 1) [31]. Under nutrition starvation and stress conditions, DGDG is exported to various extraplastidial membranes, substituting for phosphoglycerolipids [32,33,34]. Thus, MGDG and DGDG play important roles in the structural stabilization and function of membranes [35,36,37], and are fundamental in the trafficking of lipids between subcellular compartments [35].

SQDG is a negatively charged GL composed of a monoglycosyl diacylglycerol with a sulfonic acid linked in the position 6 of the monosaccharide moiety (1,2-diacyl-3-*O*-(6-sulfo-6-deoxy-α-d-glucosyl)-*sn*-glycerol [19,20,38,39]. The presence of the sulfoquinovosyl moiety is responsible for the negative charge of the sulfonic residue at physiological pH [38]. SQDG molecular species have a high content of hexadecanoic acid (16:0), octadecadienoic acid (18:2), eicosatetraenoic acid (20:4) and eicosapentaenoic acid (20:5) [19,40]. SQDG preferably adapts a cylindrical shape suitable for packing side by side in a bilayer [41]. SQDGs play important roles in the “membrane mosaic”, particularly in signaling and in the coordination between chloroplast lipids (outer envelope membrane) and cytosolic partners [41].

MGDG, DGDG and SQDG are major components of the plastid lipids [12,35,41]. MGDG constitutes the outer (20%) and the inner envelope of chloroplast and thylakoid membranes (40%–55%) [12]. DGDG represents about 15%–35% and SQDG about 2%–40% of total lipids in both chloroplast and thylakoid membranes [12]. The content of SQDG in microalgae is relatively high when compared with other green plants (2%–10%), like *Arabidopsis thaliana* [12,30,41]. Glycolipids bearing only one fatty acyl chain (lysoglycolipids) can be found in microalgae, although with low abundance, such as monoacyl monogalactolipids (MGMGs), monoacyl digalactolipids (DGMGs) and monoacyl sulfoquinovosyl lipids (SQMGs) [42]. Also glycolipids containing three galactoses (trigalactosyl diacylglycerol, TGDG) were reported in dinoflagellate glycolipidome [43]. TGDGs were previously described in the glycolipidome of the plant *Arabidopsis* [44]. Digalactosyl triacylglycerol (DGTG) and sulfoquinovosyl triacylglycerol, with a fatty acyl moiety esterified at the C-3 of the sugar unit, were described in the lipidome of cyanobacteria [20].

**Figure 2:**
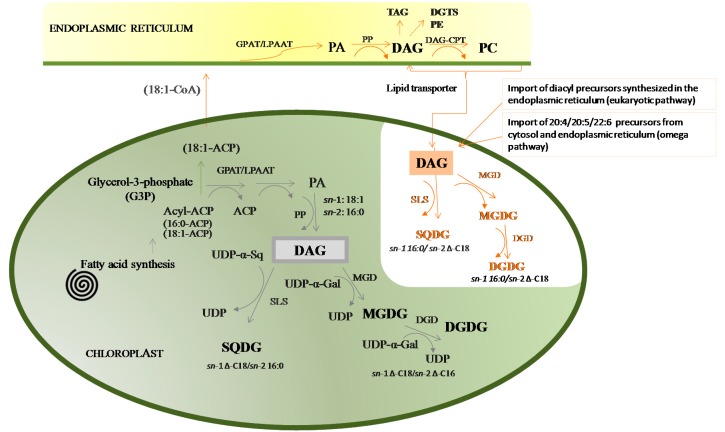
Simplified diagram of the main pathways involving the biosynthesis of glycerolipids in microalgae. The set of reactions occurring within the chloroplast are termed the chloroplastic or ‘‘prokaryotic” pathway and those that involve glycerolipid synthesis in the ER (endoplasmic reticulum) and subsequent transfer to the chloroplast constitute the endoplasmic or “eukaryotic” pathway. Orange arrows refer to the biosynthetic pathway of transport of ER-derived glycerolipids to chloroplasts. Enzymes involved in the biosynthesis of glycolipids are shown. ACP, acyl carrier protein; PA, phosphatidic acid; DAG, diacylglycerol; PC, phosphatidylcholine; MGD, MGDG syntases; DGD, DGDG synthases; UDP, uridine diphosphate galactose intermediate in the production of polysaccharides (-Gal galactose, -Sq sulfoquinovose); GPAT, glycerol-3-phosphatase acyltransferase; LPAAT, lysophosphatidic acid acyltransferase; PA, phosphatidic acid; PP, phosphatidate phosphatase; DAG-CPT, diacylglycerol synthetase-choline: diacylglycerol cholinephosphotransferase; SLS, sulfolipid synthase; SQDG, sulfoquinovosyl diacylglycerol; PE, phosphatidylethanolamine; DGTS, diacylglyceryl trimethylhomo-serine; TAG, triacylglycerol; ∆, degree of unsaturation ranging from 1 to 4 double bonds.

The roles and functions of GLs depend on their structure and composition, the coordination of which is directly dependent on biosynthetic pathways. Glycolipids are mainly synthesized in the chloroplast, within the envelope membranes of plastids, by the assembly of a glycosidic moiety to diacylglycerol (DAG) [29]. This biosynthesis is orchestrated by the activities of a panel of enzymes that coordinate the synthesis of each specific lipid, the trafficking of lipid intermediates and the catabolic pathways of lipids [35]. Specific enzymes control the type of sugar linked to the polar head, the type of fatty acid and its position in the glycerol backbone. The two major biosynthetic pathways of glycolipids in microalgae, such as in plants, are the chloroplastic or “prokaryotic” biosynthetic pathway, that occur exclusively in the chloroplast, and the endoplasmic or “eukaryotic” pathway, that starts in the endoplasmic reticulum (ER) and ends in the chloroplast (Figure 2) [45,46]. In the “prokaryotic” pathway, the biosynthesis of DAG is catalyzed by acyltransferase proteins in the inner-envelope membrane of chloroplasts, which transfer C_16_ FA to the *sn*-2 position of glycerol [10,12]. Galactolipids are further synthesized by two different galactosyltransferases (Figure 2), each transferring a galactosyl residue from uridine diphospho-galactose (UDP-Gal) to the *sn*-3 position of DAG, to synthesize MGDG, or to MGDG to form DGDG. The anomeric configuration is a β-glycosidic linkage in the first sugar and a α-glycosidic linkage in the second [27,29,36]. SQDG is produced in the chloroplast´s envelope by the assemblage of a sulfoquinovose head group from the UDP-sulfoquinovose conjugated to DAG. Thus the GLs from the “prokaryotic” pathway have typically 16:0 or 16:1 at *sn*-2 [36,41].

DAG may be derived from the ER, where it is formed during the glycerolipid biosynthetic pathway, followed by subsequent transfer of DAG moieties into the chloroplast to be used in the endoplasmic or ‘‘eukaryotic” pathway. The biosynthesis of DAGs with C_18_ FA at the *sn*-2 position, which is a signature for the ER origin of a DAG, while either C_16_ (16:0, 16:1) or C_18_ fatty acids (18:0, 18:1, 18:2 or 18:3) can be at the *sn*-1 position [36,44,45,46,47,48]. These pathways depend on the export of some of the fatty acids made in the plastids to the ER to be used in the biosynthesis of structural lipids for the non-chloroplast membranes [44,49]. The “prokaryotic” *vs.* “eukaryotic” biosynthetic pathways determine the position of the fatty acyl chains in the glycerol backbone of glycolipids, particularly concerning the fatty acids C_16_ and C_18_, at the *sn*-1 or *sn*-2 positions. In fact, in the “prokaryotic” pathway, the glycolipids have mainly C_18_ FA at the *sn*-1 position while the C_16_ are mainly at the *sn*-2, and the opposite is observed for the glycolipids synthesized by the “eukaryotic” pathways.

However, glycolipids can also include PUFAs like 20:4, 20:5 and 22:6, formed by the desaturation and elongation reactions occurring in the ER (Figure 2) and C_14_ FA that can be biosynthesized via “prokaryotic” and/or “eukaryotic” routes [29,30,49,50,51]. In the chloroplast, glycolipids can undergo little turnover by deacylation–reacylation reactions [30].

In cyanobacteria, the biosynthesis of GLs is different and it has been described that the first glycolipid formed is the monoglucosyl diacylglycerol (MGlcD) followed by a second epimerisation step converting MGlcD into MGDG [13,29]. The glycolipids MGDG, DGDG and SQDG are the predominant GLs in cyanobacteria which preferably contain C_18_ at *sn*-1 and C_16_ at *sn*-2 [13].

The existent knowledge of the membrane lipids biosynthesis was mainly based on studies obtained from the green algae model *Chlamydomonas reinhardtii* [12,36] and from the plant *Arabidopsis* *thaliana* [44,45,46,47,48], but there is still a great deal to learn in this area. Clearly, more knowledge is needed concerning the structural details of glycolipids, the biosynthesis pathways of the distinct lineages and the distinctive roles of the membrane lipids, providing fascinating fields of research. The structural complexity of polar lipids and the knowledge of the biosynthetic pathway can be improved with the new advances on high resolution and accurate mass and tandem mass spectrometry technology [14,48,52,53].

## 3. Biological Properties Associated with Glycolipids from Microalgae

Glycolipids are a class of metabolites that recently has gathered interest because of their potential biotechnological applications. Moreover, they are considered promising phytochemicals with a wide range of biological properties such as antimicrobial, anti-microfouling, antitumor promoting and anti-inflammatory [9,17,23,54,55,56]. In addition, GLs isolated from marine algae seem to have modulatory effects on oxidative stress, on the inhibition effect on the production of NO and on oxidative stress-related diseases and cancers, carrying several beneficial health effects (Table 1) [9,16,23,54].

The anti-inflammatory effect of GLs extracts from the *Porphyridium* [57], *Tetraselmis* [58] and *Nannochloropsis* [59] spp. were evaluated via inhibition of lipopolysaccharide (LPS)-induced NO production in RAW264.7 cells and on the down regulation of inducible nitric oxide synthase expression. The GLs bioactive extract of *Tetraselmis* contained MGDG (18:3/16:4) and MGDG (18:4/16:4) species, otherwise the bioactive extract of *Nannochloropsis* contained four molecular species of MGDG, namely MGDG (20:5/14:0), MGDG (20:5/16:0), MGDG (20:5/16:1) and MGDG (20:5/20:5). It has been demonstrated that polar lipids extract isolated from red algae and rich in GLs had stronger anti-inflammatory activity than pure EPA isolated from the same species, suggesting that the entire polar lipids structure contributes to anti-inflammatory activity [60]. Lipid extracts from *Porphyridium* were found to inhibit NO accumulation and may be a beneficial therapeutic strategy for the treatment of NO-mediated disorders [57]. The isolated extract was found to contain sulfolipids (five molecular species) with large amounts of palmitic acid (16:0, 26.1%), arachidonic acid (20:4, 36.8%) and eicosapentaenoic (20:5, 16.6%) acids and noticeable amounts of palmitoleic acid (16:1, 10.5%).

In order to better understand the relation between the structure and anti-inflammatory activity of sulfolipids and galactolipids, purified MGDG (18:4/16:0), DGDG (18:4/16:0) and SQDG (18:3/16:0) from thermophilic blue-green alga ETS-05 were tested in two specific *in vivo* mouse models: croton oil-induced ear oedema and carrageenan-induced paw oedema [61]. Both MGDG and DGDG showed biological activity. SQDG showed less anti-inflammatory effects. MGDG with a high content of saturated FA showed lower activity and SQDG seems to be the less potent GL [61].

Glycolipids are also considered important antitumor agents. SQDGs afford inhibitory effects on tumor cell growth [10,18,21] and are a potent inhibitor of deoxyribonucleic acid (DNA) polymerase that can lead to the death of tumor cells, especially under conditions of active proliferation [62,63]. A lipid fraction rich in SQDG isolated from *Porphyridium cruentum* showed inhibitory effects on human colon (DLD-1), breast (MCF-7), prostate adenocarcinoma (PC-3) and malignant melanoma (M4-Beu) cell lines [57]. This bioactive fraction contained SQDGs with 16:0, 16:1, 20:4 and 20:5 fatty acids. MGDG and DGDG molecular species isolated from the green alga *Chlorella vulgaris* expressed antitumor activities by the inhibition of EBV-EA activation using EBV genome-carrying human lymphoblastoid cells (Raji cells) [21]. In particular, MGDG (16:2/16:2) and MGDG (16:3/16:2) have shown a higher inhibitory effect.

Antiviral properties of the glycolipids were demonstrated on SQDG isolated from *Porphyridium purpureum* and from *Spirulina platensis*. HSV-1 was tested by a bioassay for anti-herpes simplex virus [17,62,64]. Glycolipids extracts isolated from distinct cyanobacteria containing sulfolipids and galactolipids (Table 1) had the capacity to inhibit the polymerase activity of HIV-1 RT [20,39]. The mechanism of the action of GLs remains obscure, but as was stated above, the bioactivity of GLs results from the sugar moiety, the position of the glycerol linkage to the sugar, the length and location of the acyl chain and the anomeric configuration of the sugar [65]. The antiviral activity of SQDGs may be supplied by the sulfonate group [17]. It was postulated that lipophilic groups of SQDG interact with the positive charged side of DNA polymerase. The sulfonate part of SQDG is also important in inhibiting the polymerase activity.

As well as the varied and interesting biological properties of glycolipids, they are also considered a rich source of *n*-3 PUFAs essential for human and animal nutrition, being used as food or as additives or supplements [9,23,54,66]. The *n*-3 PUFA, particularly EPA and DHA, have beneficial effects at different levels, from fetal development to cancer prevention [67]. The *n-*3 PUFAs have a key role in the prevention of cardiovascular disease, by stabilizing the atherosclerotic plaque and reducing the infiltration of inflammatory and immune cells (lymphocytes and macrophages) into the plaque [68,69]. Many chronic conditions, cardiovascular diseases, diabetes, cancer, obesity and autoimmune diseases increase with higher *n*-6 fatty-acid intake but decrease with *n*-3 fatty-acid intake [67,70]. Thus, it is recommended the optimal ratio of (*n*-6)/(*n*-3) ranges from 1/1 to 4/1, crucial to reduce the risk of many chronic diseases such as cardiovascular and neurodegenerative diseases [70]. Microalgae are rich in *n*-3 PUFAs like 18:3, 20:5 or 22:6 FA [69]. In actual fact, successful commercial examples of enriched food with PUFAs from marine algae include infant milk formulas and the production of “OMEGA” eggs [54,66,68]. The *n*-3 FA from polar-lipid rich oil of the microalgae *Nannochloropsis oculata* with krill oil in rat experiments revealed higher contents of EPA in retroperitoneal adipose tissue, suggesting that MGDGs and DGDGs from microalgae may promote the effective delivery of EPA to plasma and tissues [71]. Few of the microalgae species naturally biosynthesize docosahexaenoic acids (DHA) (e.g., *I. galbana*), but several strains are able to biosynthesize EPA 20:5, whicht is currently commercialized as a food supplement [23,68,72]. Most of GLs isolated from microalgae contain EPA, particularly Chlorophyta, Bacillariophyta and Eustigmatophyta, an essential nutrient for the production of zooplankton. EPA is used in aquaculture due to its anti-inflammatory properties and in human health due to its recognized benefits [24,26,30,56].

GLs have positive effects on the survival, growth, development, productivity and fertility of animals, boosting their health [23,66]. The beneficial properties of GLs, namely as anti-inflammatory promoters [17,61], enhance the use of microalgal biomass enriched in GLs as feed additives, namely in poultry production and aquaculture. The cosmetic and pharmaceutical industries are prominent users of GLs [8,23,66] and it is expected that the next trends in skin care may have formulas enriched with GLs, bringing direct potential benefits to the epidermis, or may use GLs as a carrier of other substances due to their emulsifying properties [8,23].

However, further studies are needed to extend the knowledge concerning the mechanism of action of these molecules as well as their distribution between microalgae species. Glycolipids from microalgae are thus compounds with high biotechnological potential for food and health applications. Due to this fact, the need arises for new research concerning the identification of each particular glycolipid signature of each distinct microalgae lineage, as well as the deviations induced by environmental and growth conditions fostering better understanding of the relation between the microalgae species/structure and associated bioactivity.

Despite of the interesting bioactive properties of GLs (glycolipids), their structure and diversity are far from being entirely recognized, preventing the full exploitation of the biotechnological potential of microalgae.

## 4. Lipidomic Approaches of Microalgae

**Figure 3:**
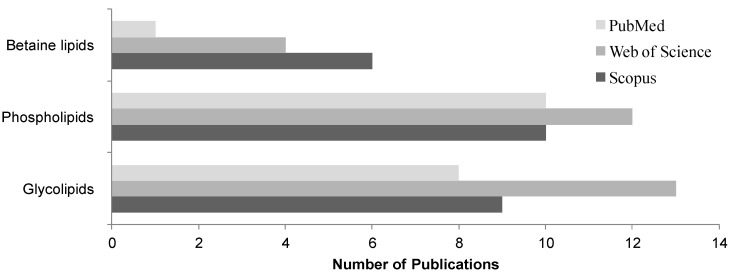
Number of original articles published between 2000 and 2015 searching the terms “glycolipids in microalgae by mass spectrometry/MS”; “phospholipids in microalgae by mass spectrometry/MS” and “betaines in microalgae by mass spectrometry/MS”. Citation analysis from *Scopus* (www.scopus.com) is represented by dark grey columns, *Web of Science* (http://apps.webofknowledge.com) database correspond to grey columns and light grey columns correspond to the search in *PubMed* (www.ncbi.nlm.nih.gov/pubmed) accessed on end of March of 2016.

Lipidomics embraces the detailed and comprehensive identification and quantification of the main molecular species of lipids [75,76]. The lipid analysis is nowadays supported by MS-based approaches that allow the profiling of the total lipid extract obtained from the algae material without chemical modification, requiring relatively small amounts of samples [75]. The glycolipidomic profiling of microalgae requires qualitative and quantitative information on the numerous individual lipid molecular species within each MGDG, DGDG and SQDG classes [32,33,52]. Scarce studies dealing with the glycolipid composition of microalgae have been performed in the past, as indicated in Figure 3 by the number of publications in the study of glycolipids in microalgae in the period 2000–2015.

The majority of the investigations on glycolipids from microalgae used very traditional methods of lipid analysis based on previous separation of polar lipid classes by TLC (thin-layer chromatography) and by silica gel on column chromatography [19,26,32,52,60,77,78,79,80]. The analysis of the fractionated classes was further performed by GC-MS analysis of the methylated fatty acids [20,21,49,69,77,78,79]. These studies based on previous separation of classes and analysis of the fatty acids profiles were the launch topics on the lipidome of marine microalgae, but lack important information about the specific molecular species. Meanwhile, different techniques based on high-resolution 1D and 2D nuclear magnetic resonance (NMR) spectroscopy [39,58,59,80,81,82] were applied for structural characterization of isolated GLs from microalgae lipid extracts. The arrival of new modern technologies on mass spectrometry led to the emergence of several works on the lipidome of microalgae, so much that over the last years almost thirty scientific reports have been published (Table 2) [14,19,24,27,28,52].

**Figure 4:**
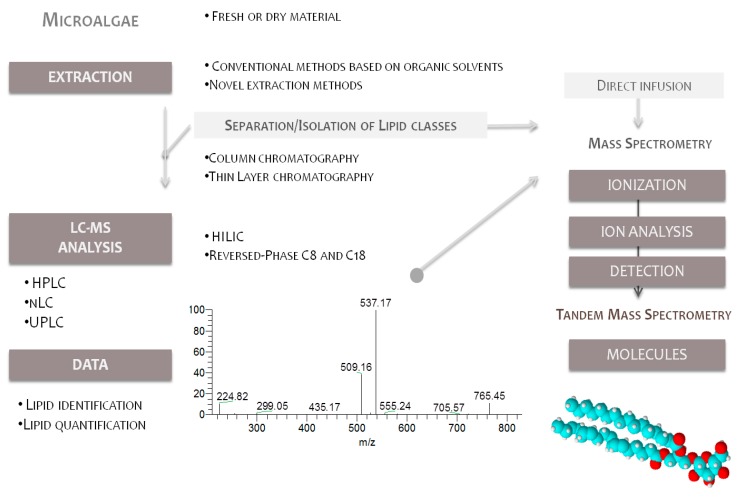
LC-MS approach in the lipidomic analysis of polar lipids from microalgae.

MS-based lipidomics platforms are a powerful potential tool in marine polar lipid research due to their relatively simple analytical process and potential for high-throughput analysis [82,83,84,85]. These advanced analytical approaches include several steps, starting with obtaining total lipid extracts using organic solvents, or nowadays green solvent approaches, followed by direct analysis by MS or by LC-MS (Figure 4). In between can be included a previous fractionation of the total lipid extract by column chromatography or TLC plates, providing the identification of GLs present in microalgae. The MS-based platform uncovers the overall profile of lipids in a specific physiological state and profile variations depending on the external conditions or alteration of homeostasis.

### 4.1. Lipid Extraction and Isolation of Glycolipids

In general, lipidomics applications require sample preparation methodology that is fast, reproducible and able to extract a wide range of analytes with different polarities compatible with the instrumental technique. Since many lipid classes consist of hydrophobic moieties (fatty acids) and polar functional groups (galactose, sulfoquinovose), most of the methods referred to in the literature are based on traditional extraction of lipids using conventional methodologies such as Bligh and Dyer [72,86] and Folch [15,87]. New methodologies for the extraction and specific isolation of polar lipids from microalgae are being studied [88,89,90,91]. Less-toxic solvents substitutes to chloroform such as ethanol, 2-ethoxyethanol, isopropanol and hexane have been investigated. The solvent to be used depends on the class of lipids to be extracted. In a recent research report, the use of 2-ethoxyethanol (2-EE) was shown to provide superior lipid recovery compared to other common extraction solvents, such as chloroform/methanol [88,92]. New green methodologies such as supercritical carbon dioxide extraction, pressurized liquid extraction or sonication [90,91,92,93] are novel methodologies to extract polar lipids from microalgae that are capable of replacing the traditional chloroform/methanol systems [86,87].

The total lipid extract can then be analyzed by LC-MS allowing identification of the glycolipids. However, the presence of other polar lipids may suppress some of the signals of glycolipids molecular species in the mass spectra. Therefore, several studies use a previous fractionation step of the total lipid extracts in order to obtain isolated fractions. The separation of the glycolipids fraction is usually carried out on chromatographic columns of silica gel, solid-phase extraction (SPE) or by thin layer chromatography (Table 2). TLC is also used for glycolipid analysis, allowing the separation of lipid classes from the total lipid extracts. Each lipid class can be further quantified by colorimetric methods followed by densitometry [26] or further characterized by GC-MS analysis of methyl ester fatty acids (FAMEs) [21,57,89]. More recently, extraction of lipids from the spots and further analysis by MS has been performed, giving the detailed structural information on the lipid molecular species within each class [32,33,52]. Quantification of GLs may be carried out by estimation of the sugar content using colorimetric methods such as anthrone and phenol sulfuric acid procedure modified for use with lipids [63,94]. These colorimetric methods can be used to estimate the total lipid content in GLs from the total lipid extracts, or to quantify the GL classes fractionated in spots of TLC plates.

### 4.2. Mass Spectrometry Analysis

Among the possible mass spectrometry-based approaches, two main strategies for the analysis of polar lipids have been used in most of the described reports: on one hand there is shotgun lipidomics, which relies on a direct infusion analysis of a total lipid extract in mass spectrometers [15,75,76], or by using LC-MS strategy [24,25]. Both methods have their advantages and disadvantages. While the shotgun approach is prone to strong ion suppression effects, this in part can be compensated for by large sample dilutions or by using internal reference compounds. Chromatography-based methods (on-line HPLC-MS, on-line UPLC-MS) avoid suppression effects, resolve problems such as the differentiation of isobaric species and allow exact lipid identification due to the chromatographic separation [85,103].

Recently, new LC-MS platforms have greatly improved the analysis of glycolipids [29,52]. The separation of lipid classes through high or ultra-performance liquid chromatography (HPLC or UPLC) is now most often used [20,24,32,52]. Usually, reversed-phase (RP C_8_ and C_18_) and hydrophilic interaction liquid chromatography (HILIC) columns are used in lipidomic approaches [85,103,104,105]. HILIC can separate lipid molecular species from different lipid classes from the complex extract of algae. However, in the majority of published works, RP has been most widely used for the analysis of complex lipids as it can distinguish polar lipids supported by their hydrophobic properties based on the number of carbons and the degree of saturation of fatty acyl substituents (Table 2). In fact, separating lipids according to the two independent molecular properties offers great opportunities towards further unscrambling the lipidome complex.

By LC-MS and MS/MS data interpretation it is possible to identify a large number of molecular species from the polar lipid classes in the total lipid extract [14,52]. The identification of the molecular ions is based on the detailed analysis of the MS spectra, and the confirmation of the identity of each molecular species is done by the analysis of MS/MS fragmentation. Unlike phospholipids, there are scarce databases including glycolipids mass spectral data [34,105,106]. LipidBlast is a database available to identify individual lipid molecular species by matching mass precursor ions and MS/MS fragmentations. However, there are several drawbacks when using this bioinformatic tool, such as the low mass accuracy of the instrument, isobaric interferences and ion suppression [105,106]. Moreover individual analysis of the fragmentation pattern of each species is usually needed in order to identify unequivocally the molecular species of glycolipids [107,108,109,110], as will be explained in the next section.

MS and LC-MS-based analytical tools are powerful for qualitative but also for quantitative analysis of the glycolipidome [15,24,25]. After the identification of molecular species and lipid classes, semi-quantitation of lipid species is achieved by the normalization of the individual molecular ion-peak intensity using an internal standard of each lipid class. The calculated ratio of analyte and internal standard is multiplied by the concentration of the internal standard to obtain the concentration of a particular analyte [85]. The sum of each peak area related with the standard representing each class gives the relative abundance of the different classes of GLs. However, the major drawback results from the unavailability and lack of stability/prohibitive price of reference standards of all glycolipids molecular species. Reviewing the lipidomics papers cited, those including quantitation used at least one internal standard per lipid class [24,25].

#### Analysis of Glycolipids by Mass Spectrometry

In the field of microalgae, great improvement to lipidomics has been made by the development of soft ionization methods such as electrospray ionization (ESI) [28]. Mass spectrometers with different analyzers, for instance ion trap (IT), orbitrap, quadrupole (Q) and time-of-flight (TOF), have been used for the structural study of polar lipids [26,27,34,38].

**Figure 5:**
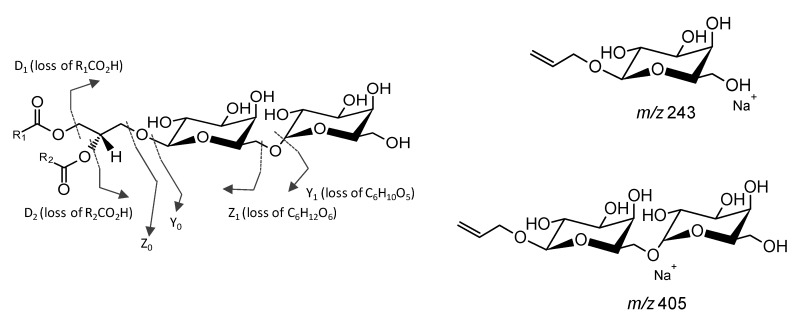
Main fragmentation pathways of digalactosyl diacylglycerol (DGDG) observed in the MS/MS spectra of the [M + Na]^+^ ions that allow the elucidation of structural features. Structures attributed to the product ions at *m*/*z* 243 and *m*/*z* 405 are depicted.

Glycolipids can be analyzed by MS in positive and negative modes attending the nature of their structural features [75,107,108,109,110]. Neutral GLs are usually identified in MS in positive mode as protonated molecules [M + H]^+^, ammonium adducts [M + NH_4_]^+^ or adducts with alkali cations (Na^+^ or Li^+^), [M + Na]^+^ or [M + Li]^+^, while SQDG are mainly detected as negative ions [M − H]^−^ [15,28,107,108,109,110,111,112]. The structural features of each molecular species are obtained by the identification of the fragmentation pattern observed in the MS/MS spectra that is unique for each species [107]. These specific fragmentation pathways allow the elucidation of fatty acyl and heading group composition of GLs [19,32]. The detailed structural features obtained from the MS/MS of GLs will be briefly described. The monogalactosyl diglycerides and digalactosyl diglycerides (MGDG and DGDG) are usually detected and fragmented as the ammonium adduct or alkali metal adducts (Figure 5) [42,48,110].

The detailed fragmentation of the precursor ion [M + Na]^+^ yields the product ion galactosyl glycerol head group [C_9_H_16_O_6_ + Na]^+^ at *m*/*z* 243 (MGDG and DGDG) and the digalactosyl glycerol head group [C_15_H_26_O_11_ + Na]^+^ at *m*/*z* 405 (DGDG) (Figure 5) [42,48,107,108,109,110]. In the case of MGDG, the neutral loss (NL) of a hexose residue (Y_1_, Figure 5) or NL of a hexose unit (Z_1_, Figure 5) in the structure of MGDG allows the confident assignment of the class. Otherwise, the loss of the hexose residues (Y_1_, Y_0_, Figure 5) or the loss of hexose units (Z_0_, Figure 5) allow to confirm the DGDG class. The information of fatty acid substituents and their position on the glycerol backbone is obtained by the identification of the product formed due to the loss of the fatty acyl moieties, described by the product ions D_1_ (loss of the R_1_COOH) and D_2_ (loss of the R_2_COOH). The regiospecificity of the two acyl chains in MGDG and DGDG is usually determined based on the ratio of the relative abundance of the loss of RCOOH from the *sn*-1 and *sn*-2 product ions attending that the loss of R_1_COOH is more abundant than the loss of R_2_COOH [97]. Also, the fragmentation of GLs observed in the MS/MS spectra as [M + NH_4_]^+^ showed typical product ions due to loss of NH_3_, loss of hexose residue (−162 Da, -Hex_res_) and hexose moiety (−180 Da, -Hex) [109,113]. Product ions formed by the combined loss of one FA and hexose yield typical acylium ions plus 74 (RCO + 74)^+^ that confirms the FA composition [110,113].

**Figure 6:**
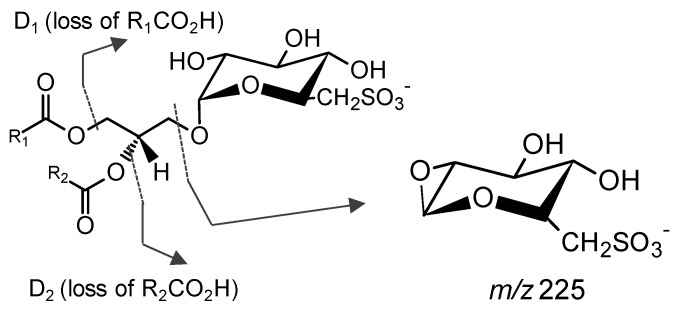
Main fragmentation pathways of SQDG observed in the MS/MS spectra of the [M − H]^−^ ion. The lines indicate the product ions formed. The product ion at *m*/*z* 225 is attributed to the sulfoquinovose head group.

SQDG preferably ionize in negative mode as [M − H]^−^ due to the strongly acidic character of the sulfonic acid. The MS/MS spectra showed a typical product ion at *m*/*z* 225 attributed to the sulfoquinovosyl anion [C_6_H_9_O_7_S]^−^, often used as a diagnostic of the presence of SQDG [24,109,112]. The neutral loss of each fatty acyl group as a ketene or free carboxylic acid (product ions D_1_ and D_2_, Figure 6) are typically observed as reasonably abundant ions. However there is not sufficient knowledge of the regioisomeric structure and specific fatty acyl groups to determine *sn*-1/*sn*-2 fatty acyl positions [97]. The product ions attributed to carboxylate anions (RCOO^−^) can also be seen [97].

### 4.3. Studies Uncovering the Lipidome of Microalgae

Microalgae mainly comprise photoautotrophic bacteria and distinct microalgae species from phyla Bacillaryophyta (diatoms), Dinophyceae (dinoflagellates), Chlorophyta and Eustigmatophyta, among others. However, only a few studies have reported the lipidomic profile of distinct microalgae. This is the case with *Chlamydomonas* sp., *Chlorella* sp., *Porphyridium purpuream*, *Nannochloropsis* sp., *Dunaliella tertiolecta*, *Isochrysis galbana*, *Chaetoceros calcitrans*, *Cyclotella meneghiniana*, *Phaeodactylum tricornutum*, *Stephanodiscus* sp*.*, *Spirulina platensis, Scenedesmus* sp*.*, *Tetraselmis chuii*, *Nitzschia closterium* and *Schizochytrium limacium*, which only recently have been investigated using MS-based approaches (Table 2). The next sections pinpoint the current research in the lipidome of Cyanobacteria, Chlorophyta, Bacyllaryophyta, Dinoflagellata, Eustigmatophyta, Prymnesiophyta and Rodophyta microalgae.

#### 4.3.1. Glycolipidomic Profiling on Cyanobacteria

Cyanobacteria are photosynthetic prokaryotes occurring in the phytoplankton. The lipidome of *Synechocystis* sp., *Scytonema* sp., *Oscillatoria* *raoi* and *Spirulina* sp. have been characterized [20,42,93]. The lipidic extract of *Synechocystis* sp. was studied by fast atom bombardment FAB-MS and MS/MS, enhancing for the first time a direct and rapid structural identification of a few glycerolipids at the molecular level [42]. The total lipids of the harvested microalgae were extracted by the method of Bligh and Dyer and the glycolipids were fractioned by two-dimensional thin-layer chromatography (2D-TLC), allowing the separation of six classes of glycolipids (14 molecular species) and two classes of phospholipids. The glycolipids of each of the spots were identified by FAB-MS as [M + Na]^+^ ions correspondent to MGDG, DGDG and SQDG with 16:0, 18:1, 18:2 and 18:3 FA and MGMG, DGMG and SQMG with 16:0 FA. Regarding *Scytonema* sp. and *Oscillatoria* *raoi* [20], besides the glycolipids MGDG, DGDG and SQDG, novel acylated glycolipids of SQDG and DGDG were assigned for the first time in the lipidome of microalgae by MS (Table 2).

The lipidome of *Spirulina* sp., the most commercialized microalgae used as food [93], was studied by LC-MS analysis of the ethanolic extract obtained using pressurized liquid extraction (PLE). This study allowed the identification of six GLs, namely MGMG (16:0), MGMG (16:2), MGMG (18:2), MGMG (18:3) and SQDG (18:3/16:0) and SQDG (18:2/16:0). The cyanobacteria were found to contain lyso-forms of glycolipids and preferably produce C_16_ and C_18_ PUFA esterified to polar lipids and do not biosynthesize EPA or DHA.

#### 4.3.2. Glycolipidomic Profiling on Chlorophyta

Green microalgae *Chlorella*, *Dunaliella* and *Clamydomonas* spp. are widely commercialized microalgae. Most of the recent published works were focused on the lipidome of *Chlorella* sp*.*, highly prized for commercial applications for food consumption and for feed [42,52,72,95,96]. The polar lipids of the *Chlorella* were analyzed by LC-MS/MS-based approaches [28,52,95]. The lipidome of *Chlorella* was fully characterized by Yao *et al.* (2015), and the MGDG (13 molecular species), DGDG (12 molecular species) and SQDG (three molecular species) were found to represent about 79% of the total polar lipids [52]. *Chlorella*´s polar lipid fraction contained a high amount of PUFA, including 16:3 (13%), 18:2 (23%) and 18:3 (21%). Regarding the lifecycle of *Chlorella*, polar lipids have the highest yield at the onset of the stationary phase, containing MGDG (32%) with the dominant components MGDG (18:3/16:3) and MDGD (18:4/18:4). SQDG corresponded to about 36% of the total extract and contained SQDG (16:1/16:0) abundant species. DGDGs (6%) mainly contained DGDG (20:5/14:0) and DGDG (20:5/16:0) [72].

GLs profile of *Chlamydomonas* were also studied by LC-MS (Table 2) [26,32,33,34,52]. The MGDG (14 molecular species), DGDG (16 molecular species) and SQDG (three molecular species) moieties represented 96% of the total polar lipids (10% of the total extract). For *Chlamydomonas*, DGDG and MGDG contained distinct isoforms with 34 carbons and 1–6 double bonds distributed in two acyl groups, which covers all of the C_16_ and C_18_ major fatty acids in *Chlamydomonas* extract.

More than 40 lipid molecular species distributed by the three subclasses MGDG, DGDG and SQDG were identified for the microalgae *Tetraselmis chuii* [28]. The detailed analysis of the tandem mass spectra of the sodium adducts of each GL molecular species allowed pinpointing the position of the two fatty acids acyl chains of MGDG and DGDG. For the 11 MGDG molecular species, the fatty acids at the *sn*-1 position were 18:1, 18:2, 18:3 and 18:4, and the fatty acids at the *sn*-2 position were 16:0, 16:1, 16:2, 16:3 and 16:4. The major molecular species identified were MGDG (18:1/16:1), MGDG (18:1/16:0), MGDG (18:4/16:4) and MGDG (18:3/16:4). For the seven DGDG molecular species, the fatty acids assigned to the *sn*-1 position were 18:1, 18:2 and 18:3 FA. In the 16 SQDG molecular species, the preferential fatty acid at the *sn*-2 position was assigned as 16:0, while the fatty acids at the *sn*-1 position included C_14–20_ fatty acids with different unsaturation and hydroxylation. The major SQDG were attributed to SQDG (18:1/16:0) and SQDG (18:3/16:0), and other SQDG molecules, such as SQDG (20:1/16:0) and SQDG (HO-18:3/16:0), were found in significant quantities [28]. Moreover, the study of the lipidome of *Scenedesmus* sp. by LC-MS allowed the identification of about 46 molecular species as MGDG (14 molecular species), DGDG (16 molecular species) and SQDG (six molecular species), which represented 67% of the total polar lipids [52]. The GLs were rich in 16:0 (31%), 16:4 (6%), 18:1 (11%), 18:2 (11%) and 18:3 (21%) and in the unusual *n*-3 fatty acid 18:4 (4%). The FA 18:4 is as effective as marine oil-derived long-chain *n*-3 fatty acids in providing the health benefits in humans associated with cardiovascular disease. In general, SQDG have preferentially saturated fatty acids (SFA) and monounsaturated fatty acids (MUFA), whereas MGDG and DGDG are mainly linked to PUFA.

Also, the commercial strain *Dunaliella* sp. [15] was profiled by means of polar lipids using MS-based approaches. Among several polar lipids, the classes MGDG (two molecular species), DGDG (six molecular species) and SQDG (four molecular species) were identified in the lipidome of *Dunaliella tertiolecta*. Interestingly, a lower number of molecular species were identified in comparison with the ones reported for the *Chlorella* or *Scenedesmus* lipidomes [52]. The analyses of the effect of the incidence of light and nitrogen deficiency was evaluated during the stationary phase of *Dunaliella tertiolecta* [15] and a significant increase of DGDG molecular species containing unsaturated C_16_ fatty acids were detected after both nitrogen starvation and high light intensity. In high light conditions, DGDG molecular species with saturated fatty acid 16:0 showed a significant decline. Nitrate deficiency did not lead to any significant changes in SQDG molecular species, while cultivation under a high light intensity resulted in an increase of SQDG (18:1/16:0) and SQDG (16:0/16:0). Under both effects, SQDG incremented in *D. tertiolecta*. SQDG has an important role in maintaining function and structure of the photosynthetic apparatus complex, especially under stress conditions such as high temperature [15]. MGDG molecular species did not exhibit variations under stress conditions.

The evaluation of the effect of nutrient limitation on *Chlorella* under *N* deprivation led to decreases in MGDG and DGDG (66%–78%) and in SQDG (40%–67%) contents [95]. MGDG, DGDG and SQDG contained 14, 16 and 20 molecular species, respectively. Analogous effect was found in the genus *Clamydomonas* sp. [32,33,34] that under *N* deprivation, showed a decrease of MGDG and DGDG species, containing 16:0, 18:1, 16:3, 16:4 and 18:3 (Table 2). The Pi starvation also induced some effect on GLs of *Chlamydomonas*, causing an increase in the amount of DGDG and SQDG species [10,33]. In response to changes in salinity, there was an increase in the amount of glycolipids in *Chlamydomonas*, mainly expressed by the increase in DGDGs content [32]. Under salt stress, there was a remarkable increase of SQDG and DGDG/MGDG ratio that is proportional to the concentration of NaCl, followed by the increase of unsaturated molecular species. DGDGs exhibit bilayer-forming properties, while MGDG are always non-bilayer forming, and the ratio DGDG/MGDG is required for the maintenance of membrane stability and functionality [32]. The GL signature of microalgae under distinct stress conditions was accomplished by using MS-approaches.

Among the distinct studies profiling the lipidome of microalgae, the reviewed studies support the fact that green algae preferably produce a typical signature and contain high unsaturated C_16_ and C_18_ FA esters bonded to glycolipids. About fifty molecular species of glycolipids were identified in the lipidome of green algae.

#### 4.3.3. Glycolipidomic Profiling on Bacillariophyta

Bacillariophyta or diatoms are single-cell eukaryotic microalgae with promising application in nanotechnology and biotechnology, including nanofabrication techniques, chemo and biosensing and control of particles in nanofluidics systems [114]. These microalgae have also potential to be used in analyzing ecological problems such as climate change, acidification and eutrophication of aqueous ecosystems [114]. Lipid profiling of distinct marine diatoms has been reported for the diatoms *Skeletonema*s sp., *Thalassiosira weissflogii*, *Stephanodiscus* sp*.*, *P. tricornutum*, *Haslea ostrearia*, *Navicula perminuta* *and Nitzschia closterium*.

Likewise, methanol and chloroform/methanol/water fractions obtained from three strains of *Skeletonemas* sp. were analyzed by LC-MS to profile the glycolipids [24]. Overall, 19 MGDGs (45%–70% of lipidome), nine DGDGs (5%–15% of lipidome) and 22 SQDGs (10%–40%) have been unequivocally identified in the three strains of *Skeletonema*s sp. The predominant molecular species of MGDG and DGDG contained 16:1, 16:2, 16:3 and 20:5 FA (Table 2) and the major molecular species of SQDG combine 14:0, 16:0, 16:1 and 16:3 FA [24]. Similar molecular species were found in *Stephanodiscus* sp. lipidome, which showed 16 MGDGs, nine 9 DGDGs and 23 SQDGs [27]. Dodson *et al.* (2013) compared the GL profiles of two centric diatoms, *Skeletonema marinoi* and *Thalassiosira weissflogii*, and the pennate diatoms, *P. tricornutum*, *H. ostrearia* and *Navicula perminuta*, characterized by ESI-MS^n^ approaches [98]. *Skeletonema marinoi*, *Thalassiosira weissflogii* and *P. tricornutum* contained MGDG and DGDG predominantly with C_16_, C_18_ and C_20_ FA. However, this was not observed within all strains and the pennate diatoms *H. ostrearia* and *Navicula perminuta* contained primarily C_18_/C_16_ or C_18_/C_18_ forms of MGDG and DGDG, indicating an unrecognized fatty acid diversity in GLs from diatoms that needs more research to be understood [98].

LC-MS was used to study the changes of the lipidome between genera and species, and also to evaluate the effect of distinct growing stages of microalgae. At the stationary phase, the glycolipidome of *P. tricornutum and C. calcitrans* enclosed abundant MGDG (18:3/16:3), MGDG (18:4/18:4), DGDG (20:5/14:0), DGDG (20:5/16:0) and SQDG (16:1/16:0) molecular species [72]. The different content of GLs allowed the differentiation of strains [72]. During the growth phase of *Nitzschia closterium*, glycolipids were found to decrease from the exponential to the stationary phase. However, at the end of the stationary phase, the levels increased due to the role of GLs in the protection of microalgae during the growth phase [99]. The molecular species MGDG (20:5/16:2), MGDG (20:5/16:3) and MGDG (16:2/16:3), DGDG (20:5/16:1), SQDGs (16:1/14:0) and lyso-SQDGs 16:0 and 16:1 are potential biomarkers of the growth cycle [99]. The examples mentioned above enhance the usefulness of MS/MS in profiling GL-rich lipids for artificial rearing of marine organisms. Based on the positional distribution of fatty acids on the individual lipid class of the diatoms, it was indicated that MGDG and DGDG were biosynthesized through the “prokaryotic” pathway exclusively within the chloroplast, because all the fatty acids linked at the *sn*-2 position in MGDG and DGDG were C_16_ FAs. Additionally, SQDG had a typical mixed biosynthetic pathway (both “prokaryotic” and “eukaryotic”) because the fatty acid at the *sn*-2 position was either C_16_ or C_18_ fatty acids. However, some pinnate diatoms were found to be able to biosynthesize C_18_/C_16_ or C_18_/C_18_ forms of MGDG and DGDG lipids [98]. Several species of diatoms were characterized by MS approaches and more than fifty molecular species are known in the signature of glycolipids. However, the distinct ability to biosynthesize C_18_ and C_20_ species is far from being elucidated. Certainly, some species of diatoms are prone to biosynthesize EPA (20:5) [24,72,98,99].

#### 4.3.4. Glycolipidomic Profiling on Dinoflagellata

Despite their importance in marine and freshwater microalgal assemblages, dinoflagellates have been the subject of few comprehensive lipid studies, particularly with respect to those lipids that comprise plastid membranes. An effort is being made to understand the differences between dinoflagellate composition in glycolipids by MS [97]. *Glenodinium sanguineum* was analyzed by LC-MS to demonstrate the correct fatty acid composition and also provide a reliable determination of the regiochemical distribution of the acyl groups on the glycerol backbone [97]. The structure of MGDG and DGDG was fully elucidated by NMR, HPLC/ESI-ITMS and by ESI-ITMS after the enzymatic hydrolysis at *sn*-1 by lipase XI, which allowed the identification of the FA position. The dinoflagellates examined were divided into two clusters based on the forms of the MGDG and DGDG profiles [100]. One group showed MGDG (18:5/18:4), MGDG (18:5/18:5), DGDG (18:4/18:4) and DGDG (18:5/18:4) as major forms, and the second one showed major MGDG (20:5/18:4), MGDG (20:5/18:5), DGDG (20:5/18:4) and DGDG (20:5/18:5) forms. The differentiation between C_18_/C_18_ and C_20_/C_18_ glycolipids indicates the predominance of different biosynthetic pathways, as well as a possible evolutionary divergence between the two dinoflagellate clusters [100]. Furthermore, these unusual octacosapolyenoic FAs seem favorable for distinguishing possible evolutionary divergence between species [100]. This knowledge was never accessed before due to the limitations of traditional methodologies, such as GC/MS analysis, which did not allow the association of each fatty acid with the molecular species of glycolipids. Moreover, the distinguishable difference between the GL profile of warm- and cold-adapted was the level of unsaturation in the *sn*-2 fatty acid of DGDG, which was found to be almost exclusively 18:5*n*-3 in cold-adapted species and a mixture of 18:5*n*-3 and 18:4*n*-3 in warm-adapted species [43]. Leblond *et al.* (2010) observed in different species of the C_20_/C_18_ peridinin-containing dinoflagellate *Pyrocystis* sp. that fatty acid modulation occurred consistently within the *sn*-2 fatty acid of DGDG according to growth temperature [101]. The modulation occurred between the 18:5*n*-3 and 18:4*n*-3 forms at the *sn*-2 fatty acid of DGDG, while the *sn*-1 fatty acid 20:5*n*-3 remained constant. Therefore, maximum unsaturation of the C_18_ *sn*-2 fatty acid of DGDG to produce 18:5*n*-3 is a key response to temperature for these algae [43,101]. Also, trigalactosyl diacylglycerol (TGDG) lipids were newly described in dinoflagellates [43].

Due to the large variety of genera lineage of dinoflagellates, much information is still unknown about glycolipids’ signature and biosynthetic pathways. Future studies are needed to determine the biosynthesis and function of TGDG in dinoflagellates and are also needed to examine the modifications on the lipidome of dinoflagellates under various growth conditions, such as temperature, with a particular interest in the species preferably producing 20:5 FA [100,101].

#### 4.3.5. Glycolipidomic Profiling on Eustigmatophyta

Regarding the Eustigmatophyta, lipidomics studies were mainly focused on *Nannochloropsis* sp. [14,52,95,102], including *Nannochloropsis oculata* and NMBluh014 and NMBluh-X strains (Table 2). All lipidomic studies of Heterokontophyta reported the MGDG, DGDG and SQDG profile, but, depending on the papers, different molecular species where detected, which is maybe due to the variation of the lipidome within different strains of microalgae. Also, the different methodologies and mass analyzers may lead to distinct information of the lipidome. He *et al.* [14] profiled the glycolipids of *Nannochloropsis oculata* and unequivocally identified 46 molecular species of MGDG, MGMG, DGDG, SQMG and SQDG. The glycolipidome contained C_14–20_ and the most abundant molecular species were MGDG (20:5/20:5), DGDG (20:5/16:0) and (20:5/16:1). *Nannochloropsis* sp. [52] was mainly composed of the molecular species SQDG (16:1/16:0), MGDG (20:5/14:0), DGDG (20:5/16:0), DGDG (20:5/16:1) and DGDG (20:5/14:0), in agreement with the results obtained for *Nannochloropsis oculata* [14]. Moreover, Li *et al.* [102] profiled two *Nannochloropsis* oceanic strains, NMBluh014 and NMBluh-X, and have discovered 30 discriminant polar lipid biomarkers providing the lipidomic differences for chemotaxonomy. The molecular composition of glycolipids of the two strains was remarkably different regarding the MGDG and SQDG species. MGDG (18:3/16:3), MGDG (20:5/14:0), SQDG (18:1/16:0) and SQDG (18:3/16:0) were only detected in NMBluh-X. Also, SQDG biomarkers containing molecular species with C_16_ FA and MGDG biomarkers containing C_16_ and C_20_ FAs were clearly detectable in NMBluh-X but there were only traces in NMBluh014. This last strain contains DGDG (18:3/16:2). The NMBluh014’s glycolipidome is remarkably different from the remaining species of *Nannochloropsis* reported until now.

Moreover, by using the MS-based approach it was possible to distinguish the taxonomic variation of Heterokont’s glycolipidome of Eustigmatophyta *Nannochloropsis* that biosynthesize glycolipids containing 20:5 FA, and the Labyrinthulomycetes *Schizochytrium* that contains trace amounts of GLs, but contains GLs bearing 22:6 fatty acyl species [52]. One of the promising applications of the MS-based strategy concerns the profiling of the lipidome under environmental stress manipulation and nutritional variations [10,95]. The response of *Nannochloropsi*s sp. to *N* deprivation [97] induced the decrease in the content of glycolipids, containing several MGDG and DGDG molecular species with abundant 20:5 FA, and 14:0 and 16:0/1 shorter FA. SQDG was the most abundant lipid in *Nannochloropsis* sp., mainly the SQDG (16:0/16:1). The MS-based approach allowed the identification of MGDG (18:3/16:3), MGDG (18:3/16:4), MGDG (18:1/16:3) and DGDG (18:2/16:0) species as important biomarkers under N deprivation. One key difference in the overall molecular profile of *Nannochloropsis* sp. is that it produces EPA (20:5), and has less effect on N deprivation within this strain.

#### 4.3.6. Glycolipidomic Profiling on Prymnesiophyta

The Haptophyta/Prymnesiophyta *I.* *galbana* was analyzed by LC-MS/MS during distinct stages of the growth cycle [72]. All types of GLs (MGDG, DGDG and SQDG) in *I. galbana* were significantly increased from the exponential phase to the end of the stationary phase. This microalgae distinguishes from most of the other categories due to its ability to biosynthesize docosahexaenoic acids (DHAs). DHA were found in MGDG and SQDG lipids at the stationary stage of *I. galbana* and a higher content of DHA-rich lipids could be obtained at the end of the stationary phase.

#### 4.3.7. Glycolipidomic Profiling on Rhodophyta

Regarding the red microalgae, only two studies were performed using MS-based approaches characterized to study the glycolipidome of *Porphyridium* sp. by the analysis of isolated fractions of total lipid extract. The extract, rich in SQDGs, was found to contain SQDG (18:2/16:0), SQDG (20:4/16:0) and SQDG (20:5/16:0) [19]. Meanwhile, the analysis of the glycolipids-rich extract of *Porphyridium aerugineum* allowed to identify five MGDG molecular species as MGDG (20:4/18:3), MGDG (20:4/16:0), MGDG (20:5/16:0), MGDG (20:5/18:3) and MGDG (20:5/20:4) [73]. In these particular cases, MS was successfully applied to identify the glycolipids on particular extracts used to evaluate the anti-proliferative and antioxidant activities [19,57,73].

## 5. Final Considerations and Perspectives

Throughout the present review paper, information has been provided as regards the significance of microalgae as a valuable reservoir of glycolipids with promising bioactive properties such as anti-viral, anti-inflammatory and antitumor promoters. These lipids have high biotechnological potential for food and health applications, and thus their structural elucidation is mandatory for understanding their structure–biological activity relationships.

Mass spectrometry-based approaches have been presented as valuable analytical tools for identification and quantification of glycolipids in routine analysis of microalgae. By using MS-based approaches, more than 20 monogalactosyldiacyl glycerolipids, 20 digalactosyl diacylglycerolipids and 35 sulfoquinovosyl diacylglycerolipids molecular species were identified. This confirms the great advantage of MS-based lipidomics for the global identification of GLs from the extracts of marine microalgae, including GL classes, fatty acyl composition and the location of fatty acids (*sn*-1 *vs.* *sn*-2). MGDGs are usually the most abundant class of GLs, accounting for about 0.1%–70% of the total lipids. DGDGs and anionic SQDGs account for about 0.02%–50% and 0.1%–40% of the total lipids, respectively. The content and composition of GLs and their fatty acids are tightly related to the state of chloroplast membranes, the taxonomic variability, the environmental stress parameters (light, salinity, temperature, contaminants) and the nutrient availability conditions. GL signature has taxonomic significance and can be used to evaluate lipid metabolism and the responsive pathways of microalgae, so far underexploited.

Microalgae present a metabolic plasticity, meaning that they can be explored and modulated by changing the cultivation conditions or even by metabolic engineering approaches for the production of GL molecular species with high added value. High-value glycolipids from microalgae raise big expectations in the context of bioeconomy as a new generation of products with enhanced nutritional and functional qualities. Future challenges include establishing the full profile of GLs at the molecular level within intra and inter taxonomy, a better understanding of the metabolic pathways of synthesis and a comprehensive screening of the bioactive compounds produced by microalgae. The biotechnological process of customised production of biomass and bioactives recovery should be optimized, fostering their potential applications as functional ingredients or extracts in final marketable products. Only the cooperation of scientists in phycology, chemistry, biology, agronomy, food and engineering is able to address these major challenges.

## Figures and Tables

**Table 1:** Glycolipids from microalgae and their potential biological activities.

Species Name	Glycolipid	Mechanism	Reference
***Antiviral***			
*Scytonema* sp. *Oscillatoria raoi* *Oscillatoria trichoides* *Oscillatoria limnetica* *Phormidium tenue*	MGDG DGDG SQDG	Inhibit HIV-1 RT activity	[20]
*Spirulina platensis*	SQDG		[62]
***Antitumor***			
*Chlorella vulgaris*	MGDG DGDG	Antitumor properties expressed by the inhibitory effects of galactolipids on *in vitro* assay of TPA-induced EBV-EA activation on Raji cells	[21]
***Anti-proliferative***			
*Porphyridium cruentum*	SQDG	Inhibition of mammalian r-DNA polymerase Inhibition of the growth cancer cell-lines on human colon (DLD-1), breast (MCF-7), prostate adenocarcinoma (PC-3) and malignant melanoma (M4 Beu) cancer cells	[57]
***Antioxidant***			
*Porphyridium cruentum*	SQDG	Inhibition of superoxide generation by activated peritoneal mono nuclear cells (PMNs)	[57]
***Anti-inflammatory***			
*Porphyridium purpureum*	SQDG	Glycolipids inhibited the NO production through the downregulation of iNOS expression	[64]
*Tetraselmis chui* *Nannocloropsis granulata* *Porphyridium aerugineum*	MGDG DGDG	Glycolipids inhibited the NO production through the downregulation of iNOS expression	[58,59,73]
*Pavlova lutheri*	SQDG MGDG DGDG	Downregulation of the production of cytokine IL-6, IL-8	[74]
*MGDG*, *DGDG*, *SQDG*	18:4/16:0 18:3/16:0	Anti-inflammatory effect on croton oil-induced ear oedema and carrageenan-induced paw oedema	[61]
***Supply n-3 PUFA***			
*Nannocloropsis* sp.	MGDG DGDG	Glycolipids in the algal oil may promote effective delivery of EPA to plasma and tissue	[71]

**Table 2:** MS-based approach on the glycolipids composition of microalgae, as reported in literature.

Species Name	MS Approach	Extraction	Abundant Molecular Species GL-R_1_/R_2_ (Total Number of Molecular Species)	GL%	Reference
***Cyanobacterium***					
*Spirulina platensis*	LC-MS*^n^* Q-TOF	Ethanol Pressurized liquid extraction (PLE)	MGMG 16:0, 16:2, 18:2, 18:3 SQDG 18:2/16:0, 18:3/16:0 (4 Lyso-MGDGs, 2 SQDGs)		[93]
*Synechocystis* sp.	Off-line TLC FAB-MS*^n^*	Bligh and Dyer	MGDG 18:1/16:0, 18:2/16:0, 18:3/16:0 MGMG 16:0 DGDG 18:1/16:0, 18:2/16:0 DGMG 16:0 SQDG 16:1/16:0, 18:1/16:0 SQMG 16:0 (4 MGDGs, 1 MGMG, 3 DGDGs, 1 DGMG, 5 SQDGs, 1 SQMG)		[42]
*Scytonema* sp.	Off-line Silica column FAB-MS	Methanol/water Chloroform/methanol	SQTG 16:0/16:0/16:0, 16:1/16:0/16:0, 18:2/16:0/16:0, 18:3/16:0/16:0 (5 SQTGs)		[20]
*Oscillatoria raoi Oscillatoria trichoides*	Off-line Silica column FAB-MS	Methanol/water Chloroform/methanol	MGDG 18:1/16:0, 18:2/16:0, 16:2/16:0 DGTG 16:1/16:0/16:0, 18:2/16:0/16:0, 18:3/16:0/16:0 DGDG 18:1/16:0, 18:2/16:0 SQDG 16:1/16:0, 16:2/16:0, 18:1/16:0 (4 MGTGs,1 DGDG, 7 MGDGs, 3 SQDGs)		[20]
***Chlorophyta***					
*Chlorella* sp.	C_8_ LC-MS*^n^* ESI-Q-TOF	Bligh and Dyer	MGDG 18:3/16:3, 18:4/18:4 DGDG 20:5/14:0, 20:5/16:0 SQDG 16:1/16:0	MDGD 32% DGDG 6.4% SQDG 36%	[72]
	RP amide column LC-MS*^n^* ESI-QqQ	Bligh and Dyer	MGDG 18:2/16:3, 18:3/16:3 DGDG 18:2/16:3, 18:3/16:3 SQDG 16:1/16:0 (14 MGDGs, 16 DGDGs, 20 SQDG)	MGDG 42% DGDG 21% SQDG 7%	[95]
	LC-MS^n^	2-Ethoxyethanol Hexane and Folch	DGDG 18:2/18:2		[42]
*Chlorella vulgaris*	Off-line SPE-Si Off-line TLC LC-MS*^n^* ESI-QqQ	Isopropanol Chloroform/methanol	MGDG ∆-C18/∆-C16 DGDG ∆-C18/∆-C16 SQDG 16:0/16:0 (13 MGDGs; 12 DGDGs, 3 SQDGs)		[52]
*Kyo-Chlorella*	Paper spray ionization-MS LTQ-Orbitrap	No extraction	MGDG 16:2/16:3, 18:3/16:3, 18:3/18:3 SQDG 18:0/16:0, 18:3/16:0, 18:3/18:3 (3 MGDGs, 3 SQDGs)		[96]
*Chlamydomonas reinhardtii*	Off-line SPE-Si TLC LC-MS*^n^* ESI-QqQ	Isopropanol Chloroform/methanol	MGDG 20:5/14:0 DGDG 18:1/16:0 SQDG 16:0/16:0 (14 MGDGs; 16 DGDGs, 6 SQDGs)		[52]
	Nano ESI-MS*^n^* LTQ	Chloroform/methanol/water	MGDG 34:7, 36:3, 36:5 DGDG 34:7, 34:6, 34:1, 34:3, 34:2 SQDG 32:0, 34:3, 34.2, 34:1 (4 MGDGs; 5 DGDGs, 4 SQDGs)		[34]
	Off line TLC MALDI-TOF	Folch *et al.*	MGDG 36:8 and 34:7 DGDG 34:6 and several 34:*n*, *n* = 1–7 SQDG 32:1 (5 MGDGs, 9 DGDGs and 2 SQDGs)		[26]
*Clamydomonas nivalis*	C_18_LC-MS*^n^* Q-TOF	Bligh and Dyer	MGDG 18:3/16:4, 18:3/16:3 DGDG 18:3/16:4, 18:1/16:3, 18:2/16:0 SQDG 16:0/16:0, 16:0/18:3 (2 MGDGs, 5 DGDGs, 2 SQDGs) MGDG 18:3/16:2, 18:3/16:3, 18:1/16:2 DGDG 18:3/16:0, 18:1/16:3, 18:2/16:0, 18:1/16:0 SQDG 16:0/16:0, 16:0/18:3, 16:0/18:2, 16:0/18:1 (3MGDGs, 4 DGDGs, 4 SQDGs)	MGDG 41% DGDG 13% SQDG 11%	[32] [33]
*Dunaliella tertiolecta*	ESI-MS*^n^* LTQ	Folch *et al.*	MGDG 18:3/16:3 DGDG 18:3/16:4, 18:3/16:3, 18:3/16:2, 18:4/16:0 SQDG 16:0/16:0, 18:3/16:0 2 MGDGs, 6 DGDGs, 4 SQDGs)		[15]
*Scenedesmus* sp.	Off-line SPE-Si Off-line TLC LC-MS*^n^* ESI-QqQ	Isopropanol Chloroform/methanol	MGDG ∆-C18/∆-C16 DGDG ∆-C18/∆-C16 SQDG 16:0/16:0 (14 MGDGs; 16 DGDGs, 6 SQDGs)		[52]
*Tetraselmis chuii*	C_18_ LC-MS*^n^* ESI-Q-TOF	Bligh and Dyer	MGDG 18:1/16:1, 18:1/16:0, 18:4/16:4, 18:3/16:4 DGDG 18:1/16:0, 18:2/16:0, 18:3/16:0 SQDG 18:1/16:0, 18:3/16:0 (11 MGDGs, 7 DGDGs, 16 SQDGs)		[28]
***Bacillariophyta***					
*Chaetoceros calcitrans*	C_8_ LC-MS*^n^* ESI-Q-TOF	Bligh and Dyer	MGDG 18:3/16:3, 18:4/18:4 DGDG 20:5/14:0, 20:5/16:0 SQDG 16:1/16:0	MGDG 8% DGDG 4% SQDG 26%	[72] [97]
*Cyclotella meneghiniana*	Off line TLC MALDI-TOF	Folch *et al.*	MGDG 36:6 and 32:6 DGDG 36:7 and 32:1 SQDG 32:1 (5 MGDGs, 9 DGDGs, 2 SQDGs)		[26]
*Haslea ostrearia*	Off-line Silica Column ESI-LTQ-MS*^n^*	Bligh and Dyer	MGDG 18:3/16:3, 18:3/16:2, 18:2/16:3 DGDG 18:3/18:3, 18:3/16:0 (19 MGDGs, 5 DGDGs)		[98]
*Navicula perminuta*	Off-line Silica Column ESI-LTQ-MS*^n^*	Bligh and Dyer	MGDG 18:2/16:0, 18:4/16:0, 20:5/16:3 DGDG 18:3/16:3 (3 MGDGs, 1 DGDGs)		[98]
*Phaeodactylum tricornutum*	C_8_ LC-MS*^n^* ESI-Q-TOF	Bligh and Dyer	MGDG 18:3/16:3, 18:4/18:4, 16:1/16:0 DGDG 20:5/14:0, 20:5/16:0 SQDG 16:1/16:0 (21 MGDGs, 9 DGDGs)	MGDG 5.1% DGDG 3.7% SQDG 26%	[72]
	Off-line Silica Column ESI-LTQ-MS*^n^*	Bligh and Dyer	MDGD 20:5/16:2, 20:5/16:3 DGDG 16:1/16:1, 16:1/16:0, 20:5/16:1 (20 MGDGs, 9 DGDGs)		[98]
*Nitzschia closterium*	C_8_ LC-MS*^n^* ESI-Q-TOF	Bligh and Dyer	MGDG 20:5/16:2, 20:5/16:3, 16:2/16:3 DGDG 20:5/16:2 SQDG 16:1/14:0 Lyso SQDG 16:0, 16:1 (4 MGDGs, 1 DGDG, 3 SQDGs, 3 Lyso-SQDGs)		[99] [25]
*Skeletonema* sp.	C_18_ LC-MS*^n^* ESI-Q-TOF	Bligh and Dyer	MGDG 16:3/16:3, 20:5/16:1, 20:5/16:3 DGDG 20:5/16:1, 20:5/16:2, 16:1/16:1 SQDG 14:0/14:0, 14:0/16:0, 14:0/16:1, 14:0/16:3 (19 MGDGs, 9 DGDGs, 22 SQDGs)	MGDG 45-71% DGDG 11-49% SQDG 11-40%	[24]
	Off-line Silica Column ESI-LTQ-MS*^n^*	Bligh and Dyer	MGDG 16:1/16:3, 16:2/16:2 DGDG 20:5/16:1 (3 MGDGs,1 DGDGs)		[98]
*Stephanodiscus* sp.	C_18_ LC-MS*^n^* ESI-Q-TOF	Bligh and Dyer	MGDG 16:2/16:0, 16:0/16:1 DGDG 20:5/16:1, 16:1/16:0 SQDG 14:0/16:0, 16:0/16:1, 16:2/16:2, 20:5/18:4 (16 MGDGs, 9 DGDGs, 23 SQDGs)	MGDG 68% DGDG 6.3% SQDG 21%	[27]
*Thalassiosira weissflogii*	Off-line Silica Column ESI-LTQ-MS*^n^*	Bligh and Dyer	MDGD 14:0/16:1, 16:0/16:3 DGDG 20:5/16:2 (5 MGDGs, 1 DGDGs)		[98]
***Dinoflagellata***					
*Dinoflagellate* spp.	Off-line Silica Column ESI-LTQ-MS*^n^*	Bligh and Dyer	MGDG 18:5/18:4, 18:5/18:5 DGDG 18:4/18:4, 18:5/18:4 (4 MGDG, 4 DGDG) MGDG 20:5/18:4, 20:5/18:5 DGDG 20:5/18:4, 20:5/18:5 (4 MGDGs, 4 DGDGs)		[100]
	Off-line Silica Column ESI-LTQ-MS^n^	Bligh and Dyer	MGDG 18:5/18:4, 18:4/18:4, 18:5/18:5 DGDG 18:4/18:4, 18:5/18:4, 18:5/18:5, 18:1/14:0 TGDG 18:1/14:0, 18:1/16:0, 18:1/18:1 (3 MGDGs, 4 DGDGs, 3 TGDGs)		[43]
*Glenodinium sanguineum*	C_18_ LC-MS*^n^* ESI-Q-IT		MGDG 20:5/∆-C18, 20:5/∆-C16, ∆-C18/∆-C16, ∆-C18/∆-C18 DGDG ∆-C18/∆-C16, ∆-C18/∆-C18 (10 MGDGs,11 DGDGs)		[97]
*Pyrocystis* sp.	Off-line Silica Column ESI-LTQ-MS*^n^*	Bligh and Dyer	MGDG 20:5/18:5, 20:5/18:4 DGDG 20:5/18:5, 20:5/18:4 TGDG 18:1/14:0, 22:6/16:0 (2MGDGs, 2DGDGs, 2 TGDGs)		[101]
***Eustigmatophyta***					
*Nannochloropsis oculata*	C_8_ LC-MS*^n^* ESI-Q-TOF	Bligh and Dyer	MGDG 18:3/16:3, 18:4/18:4 DGDG 20:5/14:0, 20:5/16:0 SQDG 16:1/16:0	MGDG 17% DGDG 12% SQDG 6.3%	[72]
	nLC-MS*^n^* ESI FT-ICR	Chloroform/methanol	MGDG 20:5/20:5 DGDG 20:5/16:0, 20:5/16:1 SQDG 16:1/16:0 (13 MGDGs, 20 DGDGs, 33 SQDGs		[14]
*Nannocloropsis oceanica*	C_8_ LC-MS ESI-Q-TOF	Bligh and Dyer	MGDG 20:5/14:0, 20:5/16:0, ∆-C16/∆-C16, ∆-C18/∆-C16 DGDG 20:5/16:0, ∆-C18/∆-C16 SQDG ∆-C16/∆-C16, 20:5/16:0, (6 MGDGs, 4 DGDGs, 7 SQDGs)		[102]
*Nannochloropsis* sp.	Off-line SPE-Si Off-line TLC LC-MS*^n^* ESI-QqQ	Isopropanol Chloroform/methanol	MGDG 20:5/14:0, DGDG 20:5/14:0, 20:5/16:0, 20:5/16:0 SQDG 16:1/16:0 (14 MGDGs; 16 DGDGs, 6 SQDGs)		[52]
	C_8_ LC-MS*^n^* ESI-Q-TOF	Bligh and Dyer	MGDG 18:3/16:3, 18:4/18:4 DGDG 20:5/14:0, 20:5/16:0 SQDG 16:1/16:0	MGDG 13% DGDG 5.6% SQDG 18%	[72]
	LC-MS^n^ ESI-QqQ	Bligh and Dyer	MGDG 20:5/14:0 DGDG 20:5/14:0 SQDG 18:3/16:0 (14 MGDGs, 16 DGDGs, 20 SQDGs)	MGDG 42% DGDG 14% SQDG 39%	[95]
	Paper spray ionization LTQ-Orbitrap-MS*^n^*	No need of extraction	MGDG 32:3, 32:5, 34:2, 34:6 SQDG 30:0, 32:1, 34:3, 36:6 (4 MGDGs, 4 SQDGs)		[96]
***Labyrinthulomycetes***					
*Schizochytrium limacinum*	Off-line SPE-Si Off-line TLC LC-MS*^n^* ESI-QqQ	Isopropanol Chloroform/methanol	MGDG 22:6/16:0 DGDG 18:1/18:0 SQDG 16:1/16:0 (14 MGDGs; 16 DGDGs, 6 SQDGs)		[52]
***Rhodophyta***					
*Porphyridium purpuream*	Off Line TLC MALDI-QIT-TOF-MS*^n^*	Dichloromethane/methanol	SQDG 18:2/16:0, 20:4/16:0, 20:5/16:0 (3 SQDGs)		[19]
*Porphyridium aerugineum*	Off-line Silica Column LC-Q-MS*^n^*	Methanol Ethyl acetate	MGDG 20:4/18:3, 20:4/16:0, 20:5/16:0, 20:5/18:3, 20:5/20:4 (5 MGDGs)		[73]
***Prymnesiophyta***					
*Isochrysis galbana*	C_8_ LC-MS*^n^* ESI-Q-TOF	Bligh and Dyer	MGDG 18:3/16:3, 18:4/18:4, 22:6/16:0 DGDG 20:5/14:0, 20:5/16:0 SQDG 16:1/16:0	MGDG 37% DGDG 11% SQDG 41%	[72]

MGDG, monogalactosyl diacylglycerol; DGDG, digalactosyl diacylglycerol; TGDG, trigalactosyl diacylglycerol; DGTG, digalactosyl triacylglycerol, with one of the fatty acids linked to the sugar moiety; MGMG, monogalactosyl monoacylglycerol; SQDG, sulfoquinovosyl diacylglycerol; SQTG, sulfoquinovosyl triacylglycerol, with one of the fatty acids linked to the sugar moiety; SQMG, sulfoquinovosylmonoacylglycerol; C:N, total number of the carbons of the fatty acids: total number of double bonds; ∆, degree of unsaturation ranging from 1 to 4 double bonds; TLC, Thin Layer Chromatography; SPE, Solid-Phase Extraction; Si, Silica; FAB, Fast Atom Bombardment; LC-ESI/FT-ICR-MS, Liquid Chromatography-Electrospray Ionization Fourier Transform Ion Cyclotron Resonance Mass Spectrometry; UPLC, Ultra Performance Liquid Chromatography; LTQ, Linear Ion Trap; Q-IT, Quadrupole-Ion Trap; QqQ, Triple Quadrupole; Q-TOF, Quadrupole-Time of Flight; MALDI, Matrix-Assisted Laser Desorption Ionization.
